# Potentiation of cancer immunogenicity by targeting PARP

**DOI:** 10.1136/jitc-2024-011056

**Published:** 2025-06-24

**Authors:** Dominik Humer, Victoria Klepsch, Gottfried Baier

**Affiliations:** 1 Cell Genetics, Medical University of Innsbruck, Innsbruck, Tirol, Austria

**Keywords:** Immunotherapy, Tumor microenvironment - TME, Immunosuppression

## Abstract

A team of scientists led by Quigley Goa demonstrates that Poly(ADP-ribose) polymerase (PARP) inhibitors (PARPi) can induce tumor cell death in a manner that allows immune cells to better recognize and attack the tumor. Specifically, PARPi are approved for the treatment of tumors with homologous recombination repair defects. Due to their pre-existing DNA repair defects, PAPRi appear to be a pharmacological tool to induce immunogenic cell death (ICD). Remarkably, therefore, both increased tumor neoantigen generation and reprogramming of the tumor immune microenvironment to an immunostimulatory state antagonize impending immunosuppression and consequently promote enhanced antitumor immunity. This finding strongly supports PARPi targeting as a promising approach to alleviate intratumoral effector T cell immune dysfunction, particularly in the context of immunotherapy resistance. In conclusion, this well-defined relationship between PARPi-based chemotherapy and ICD of tumor cells may offer substantial potential as a valuable sensitizer for future combinatorial cancer immunotherapy, which together with immune checkpoint therapy, but potentially also with others including cancer vaccines, is likely to be more effective against defined solid tumors and better promote host-protective cancer immune control (see related article by Xia *et al*, 2024).

 Poly(ADP-ribose) polymerase (PARP) plays an indispensable role in maintaining genome integrity and facilitating DNA repair. Specifically, PARP inhibitor (PARPi)-induced DNA damage in clinical tumor cells promotes genomic instability, which subsequently leads to the generation of tumor neoantigens (novel tumor antigenic epitopes derived from somatic mutations), which can significantly increase the mutational burden of tumors.^1 2^ In parallel, PARPi-induced DNA damage has been shown to lead to the accumulation of cytosolic DNA fragments, which in turn activate the cyclic GMP-AMP synthase-stimulator of interferon genes (cGAS-STING) pathway and subsequently the production of type I interferons and other proinflammatory cytokines.^3^ Activation of the cGAS/STING pathway promotes antitumorigenic macrophage polarization and enhances antigen presentation by dendritic cells, thereby promoting intratumoral T cell infiltration and cytotoxic effector CD8^+^ T cell priming. In addition, PARPi have been shown to induce cell death in tumor cells, particularly those with homologous recombination repair defects.^1^,^3^

Quigley Goa and colleagues defined a gasdermin (GSDM) D/E-mediated immunogenic cell death (ICD) process of ovarian tumors in vitro and in situ on PARPi treatment.^4^ In both a phase II clinical trial and mechanistic studies in murine models, PARPi have been shown to induce GSDM-mediated pyroptosis of tumor cells as a specific and potent mode of ICD.^5^ The principle behind ICD is that GSDM-mediated pyroptosis initiates a potent inflammatory cascade to heat the tumor immune microenvironment (TIME) and tumor-draining lymph nodes and boost the tumor-targeting immune response,^5^ thereby contributing to tumor immune control by the host’s endogenous immune system (see schematic cartoon in figure 1). The underlying mechanism has been biochemically defined as a tumor-intrinsic cascade involving NF-κB-TNFα-TNFR-caspase 8-caspase 3-GSDMD/E initiated by PARPi-induced DNA damage.^4^ The cleavage of GSDMD and E, respectively, results in the generation of pore-forming NH2-terminal GSDMD and E fragments known to trigger the release of damage-associated molecular patterns such as calreticulin, high mobility group box 1 protein, and ATP. Subsequently, innate immune cells are recruited and activated, resulting in enhanced antigen cross-presentation of tumor neoantigens. Such an inflamed immune phenotype is well established to enrich CD8^+^ T cells within intratumoral areas. Together with an enhanced upregulation of major histocompatibility complex class I on tumor cells, this is leading to the expansion of non-self tumor neoantigen-specific T cell receptor (TCR) clones and enhanced CD8^+^ T cell-mediated tumor cell killing.

**Figure 1:**
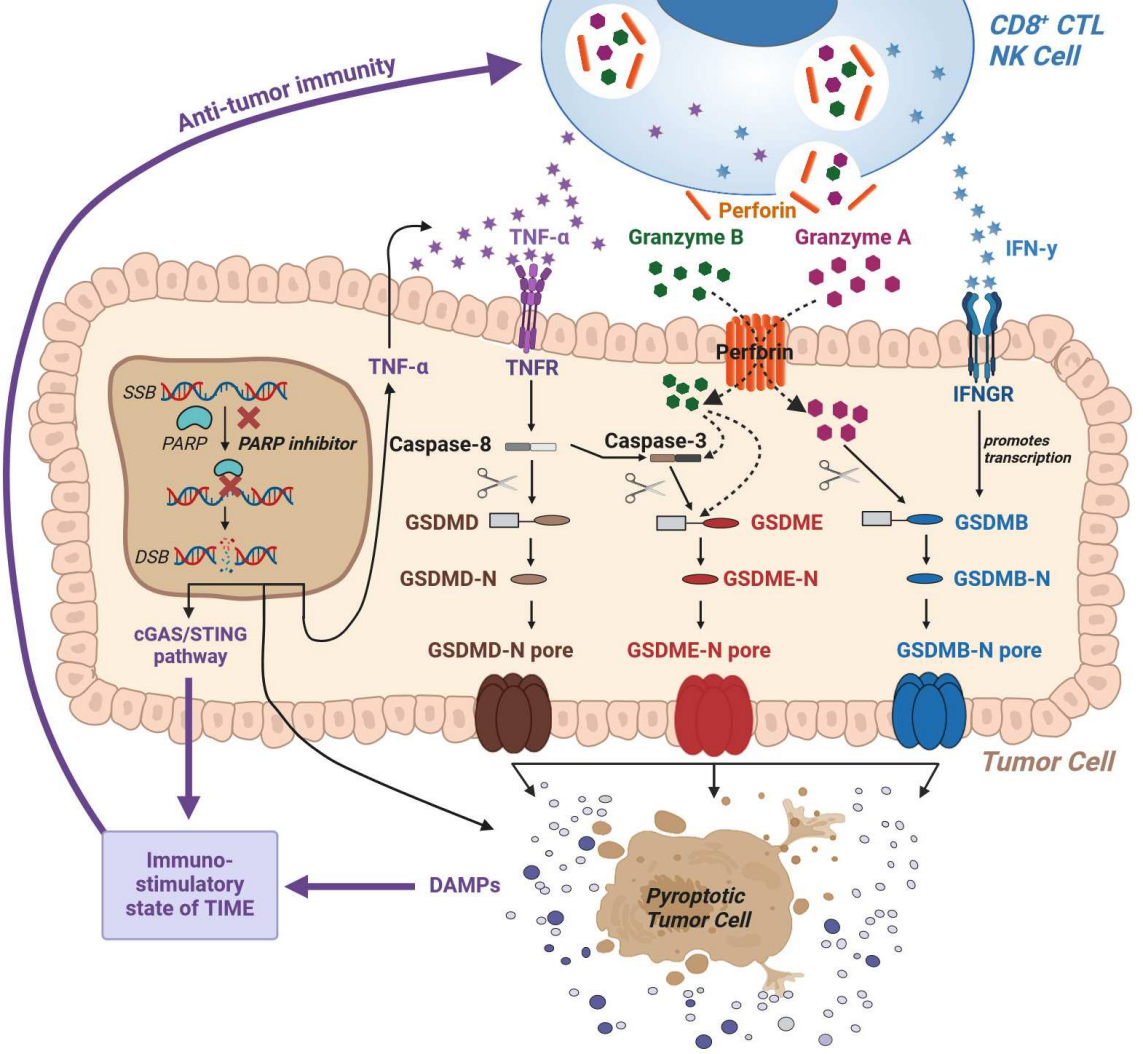
Mechanism of action of PARP inhibitors to induce pyroptosis and antitumor immunity: PARP inhibition, which augments the ability of the immune system to recognize and attack tumor cells, has emerged as a promising therapeutic strategy in cancer treatment. Mechanistically, PARPi block the single-strand break (SSB) DNA base excision repair pathway and subsequently promote the accumulation of double-strand breaks (DSB), leading to the accumulation of DNA damage.^2 3^ The resulting genomic instability contributes to the mutational burden of the tumor. In addition, the cGAS-STING pathway, a key innate immune activation pathway, is activated by the accumulation of cytosolic DNA due to unrepaired DNA damage.^4^ Subsequently, the induced inflammatory cascade recruits immune cells such as dendritic cells and T cells to the TIME. In addition, as shown in the above-mentioned study,^1^ PARPis are also able to induce an immune-stimulated form of inflammatory tumor cell death through caspase-mediated cleavage of gasdermin proteins, known as pyroptosis. PARPi triggers a tumor-intrinsic cascade involving NF-κB-mediated upregulation of TNFα. TNFα, through its receptor TNFR, induces the activation of caspase-3 via caspase-8. Activated caspases cleave the NH2-terminal domain of GSDMD and E, respectively, resulting in the generation of pore-forming N-GSDMD and E fragments. Danger-associated molecular patterns (DAMPs) released through the pyroptotic pores of dying tumor cells then further activate the innate immune system, ultimately turning a “cold" immune deserted environment into an immune-inflamed TIME as a hallmark of superior antitumor immunity. In parallel, dying tumor cells release tumor antigens for capture and presentation by activated dendritic cells. Importantly, as a major beneficial consequence of the resulting shift towards an immunostimulatory state of TIME, robust cross-priming of non-self tumor neoantigens to the patient's adaptive immune system is facilitated. Since tumor-reactive CD8⁺ T cells and NK cells themselves have been shown to promote tumor cell pyroptosis via the granzyme A/GSDMB and granzyme B/GSDME axes, respectively,^10^ the PARPi-triggered tumor antigen reactive T cell response is likely to induce a positive feed-forward loop towards further tumor antigen release. Coupled with an immunostimulatory state of TIME, the resulting shift in high ICD rates will continuously facilitate effective cross-priming of neoantigens to the patient's immune system. PARPi-based targeted therapy regimens, especially when used in combination with additional immunotherapy, may be able to achieve host-protective immune control of tumors. cGAS, cyclic GMP-AMP synthase; GSDMD, gasdermin D; ICD, immunogenic cell death; IFN-g, interferon gamma; IFNGR, interferon gamma receptor; NK, natural killer; PARP, poly(ADP-ribose) polymerase; PARPi, PARP inhibitors; STING, stimulator of interferon genes; TIME; tumor immune microenvironment; TNFα, tumor necrosis factor α; TNFR, tumor necrosis factor receptor.

The work of Quigley Goa and colleagues thereby mechanistically defines the mode of action how PARPi treatment can directly promote ICD of tumor cells and subsequently induce an immune-permissive TIME that enhances tumor neoantigen-specific immune response as a key issue of increased tumor immunogenicity by PARPi treatment, ultimately making tumors more visible to the patient’s immune system. Indeed, an increase in neoantigen-specific TCR clones and enhanced TCR clonal expansion following PARPi treatment in preclinical models were demonstrated. This result was also confirmed by TCR-seq analysis of ovarian cancer samples before and after monotherapy with PARPi niraparib in a prospective clinical trial.^4^ The authors thus rightly conclude that PARPi-induced pyroptosis improves adaptive immune cell effector functions and contributes to superior adaptive antitumor immune control.

Along this line of argumentation, it is interesting to note that the induction of pyroptosis is thought to be rate-limited by membrane repair as an intrinsic tumor cell resistance mechanism.^6^ Therefore, and because pyroptosis is antagonized by membrane self-sealing and/or active repair of GSDM pore formation, only a particularly robust imbalance between the pore-forming property of the N-terminal fragments of GSDM proteins and the counterbalancing repair mechanisms of plasma membrane damage appears able to robustly promote ICD. In theory, therefore, an intriguing mechanistic concept could be that PARPi modify tumor cells to become more susceptible to CD8^+^ T cell and NK cell killing during the lytic attack mediated by their cytotoxic granule exocytosis, which in turn intensifies GSDM-induced ICD signaling and generates a positive feed-forward loop towards augmented tumor cell immunogenicity (figure 1).

Of note, although the immunogenic effects of PARP inhibition create a favorable environment for immunotherapy to work more effectively, PARPi-induced cGAS/STING activation has also been discussed to exert immunosuppressive effects such as upregulation of PD-L1 on tumor cells.^3 7^ This phenomenon may represent the scientific rationale for the specific susceptibility of PARPi-treated tumor cells to PD-L1 blockade, thereby providing remarkable efficacy by combining these modalities in therapy. In fact, additive antitumor activity has been observed in preclinical and clinical trials when PARPi chemotherapy is combined with immune checkpoint therapy (ICT), such as PD-(L)1 blockade.^8 9^

Research has shown that PARPi can be used to selectively target defined tumors harboring breast cancer type 1 susceptibility protein 1 or 2 (BRCA1/2) mutations. Consistently, inhibition of the major DNA damage response-associated PARP, PARP1, is known to be particularly lethal to tumor cells with homologous recombination deficiencies, especially those based on defects in the BRCA1/2-dependent pathway. These are the circumstances that allow healthy cells to survive PARPi treatment.^1^,^3^ Of note, ovarian, breast, pancreatic and prostate cancers have germline mutations in BRCA1/2, making these defined tumors particularly susceptible to PARPi.^1^,^38 9^ Of note, PARPi administration appears to be associated with manageable toxicity in the clinic, despite observed adverse events including mild to moderate hematologic and gastrointestinal reactions and fatigue.^1 2 8^ Based on durable responses to PARPi-based therapy in preclinical settings, clinical approval has currently been granted for the use of potent PARP 1 and 2 inhibitors such as olaparib (ie, NCT02734004 and NCT02484404) and niraparib (ie, NCT02657889), that provide the critical proof-of-concept for the high antitumor potential of PARPi regimens.^8^ Notwithstanding these remarkable results, however, the definition of reliable predictive biomarkers for patient stratification will remain critical to make these agents more broadly applicable to patients with cancer.^9^

In conclusion, the clinical relevance of this concept of PARPi promoting inflammation and enhancing tumor immunogenicity based on the established functional link between PARPi administration to increase DNA damage and pyroptotic tumor cell death within the TIME in ovarian cancer, as now mechanistically detailed in the aforementioned study,^4^ warrants the investigation of PARPi in additional tumor entities. This may extend the benefits of PARPi monotherapy to a broader cancer patient population. In particular, data from ongoing clinical trials may validate the concept of PARPi combination therapy as an option to improve the clinical benefit of ICT in the non-responder cohort of patients.^8 9^

## References

[1] Muzzana M, Broggini M, Damia G (2025). The Landscape of PARP Inhibitors in Solid Cancers. Onco Targets Ther.

[2] Baradács I, Teutsch B, Váradi A (2024). PARP inhibitor era in ovarian cancer treatment: a systematic review and meta-analysis of randomized controlled trials. J Ovarian Res.

[3] Sen T, Rodriguez BL, Chen L (2019). Targeting DNA Damage Response Promotes Antitumor Immunity through STING-Mediated T-cell Activation in Small Cell Lung Cancer. Cancer Discov.

[4] Xia Y, Huang P, Qian Y-Y (2024). PARP inhibitors enhance antitumor immune responses by triggering pyroptosis via TNF-caspase 8-GSDMD/E axis in ovarian cancer. J Immunother Cancer.

[5] Yu P, Zhang X, Liu N (2021). Pyroptosis: mechanisms and diseases. Signal Transduct Target Ther.

[6] Rühl S, Shkarina K, Demarco B (2018). ESCRT-dependent membrane repair negatively regulates pyroptosis downstream of GSDMD activation. Science.

[7] Grabosch S, Bulatovic M, Zeng F (2019). Cisplatin-induced immune modulation in ovarian cancer mouse models with distinct inflammation profiles. Oncogene.

[8] Musacchio L, Cicala CM, Camarda F (2022). Combining PARP inhibition and immune checkpoint blockade in ovarian cancer patients: a new perspective on the horizon?. ESMO Open.

[9] Drew Y, Zenke FT, Curtin NJ (2025). DNA damage response inhibitors in cancer therapy: lessons from the past, current status and future implications. Nat Rev Drug Discov.

[10] Zhou Z, He H, Wang K (2020). Granzyme A from cytotoxic lymphocytes cleaves GSDMB to trigger pyroptosis in target cells. Science.

